# Hyperspectral imaging using the single-pixel Fourier transform technique

**DOI:** 10.1038/srep45209

**Published:** 2017-03-24

**Authors:** Senlin Jin, Wangwei Hui, Yunlong Wang, Kaicheng Huang, Qiushuai Shi, Cuifeng Ying, Dongqi Liu, Qing Ye, Wenyuan Zhou, Jianguo Tian

**Affiliations:** 1 The Key Laboratory of Weak-Light Nonlinear Photonics, Ministry of Education, Teda Applied Physics Institute and School of Physics, Nankai University, Tianjin 300071, China

## Abstract

Hyperspectral imaging technology is playing an increasingly important role in the fields of food analysis, medicine and biotechnology. To improve the speed of operation and increase the light throughput in a compact equipment structure, a Fourier transform hyperspectral imaging system based on a single-pixel technique is proposed in this study. Compared with current imaging spectrometry approaches, the proposed system has a wider spectral range (400–1100 nm), a better spectral resolution (1 nm) and requires fewer measurement data (a sample rate of 6.25%). The performance of this system was verified by its application to the non-destructive testing of potatoes.

The hyperspectral imaging (HSI) technique has been used widely and successfully in resource assessment, environmental monitoring, disaster warning and other remote sensing domains^1^,^2^,^3^,^4^,^5^. The traditional colour camera collects the red, green and blue (RGB) spectral channels, whereas the HSI system is able to acquire several hundred continuous spectral channels from a given scene^6^. A large number of hyperspectral images allow quantitative analysis with high levels of accuracy and reliability^7^,^8^. The HSI systems currently available face various problems, such as low speed, high cost and complex structure^9^,^10^. In recent years, to improve the speed, several research groups have aimed to reduce the sampling rate or increase the number of sampling channels^11^,^12^,^13^,^14^,^15^. For example, a fast spectral reflectance recovery system has been presented that is capable of taking multi-spectral measurements as fast as 100 Hz by using a DLP projector^16^. However, the sheer quantity of data still prevents further improvement of the speed. The general hyper-spectral images are very sparse in terms of space and spectrum; therefore, in theory, a data cube can be reconstructed using very little data. Based on a priori information, the hyperspectral data can be compressed and collected effectively by utilising compressive sensing (CS) theory^17^,^18^,^19^,^20^. Consequently, the development of single-shot compressive spectral imaging architectures mitigate the trade-offs between spectral resolution measurement acquisition time^21^,^22^. Furthermore, multi-shot compressive spectral imaging approaches have been achieved based on a single-pixel imaging technique, which is the first implementation of CS^23^,^24^,^25^,^26^. A high spectral resolution has benefited from the high performance of a fibre spectrometer with a single detector^27^. Although the systems that have adopted dispersion devices (prisms, gratings, or filter wheels) have promoted the development of the compressive hyperspectral imager greatly, the issues of light acquisition, time scanning and other technical barriers are still difficult to overcome^28^,^29^,^30^,^31^,^32^,^33^. As an alternative spectral imaging method, Fourier transform spectral imaging (FTSI) technology has the advantages of high levels of detection sensitivity and light throughput. The present FTSI methods use point-by-point scanning with a single detector or capture images one at a time through array detectors^34^,^35^. This method either takes a long time or sacrifices detection sensitivity. In some single-pixel hyperspectral imaging systems, Fourier transform techniques are used as structured light modulation and signal temporal modulation to exceed the diffraction limit and multiplex 3D data^36^,^37^,^38^,^39^,^40^. However, the advantages of a Fourier transform in spectral modulation are far from being explored.

To overcome the above-described limitations and provide the corresponding advantages, a hyperspectral imaging system using the single-pixel Fourier transform technique (HSI-SPFT) is presented in this paper. Spatial-spectral modulation was realised by introducing the interference device into a single-pixel architecture. We aimed to compare our system with current spectral imaging systems (regarding its spectral resolution, spectral range and light throughput) and to determine the accuracy of spectral information using sub-pixel sampling in an effort to enable this HSI system to satisfy industrial demands. The performance of the presented system was validated by both simulation and experiment. A non-destructive testing of potatoes was conducted using the proposed system.

## Results

### Simulation and the results

**Figure 1:**
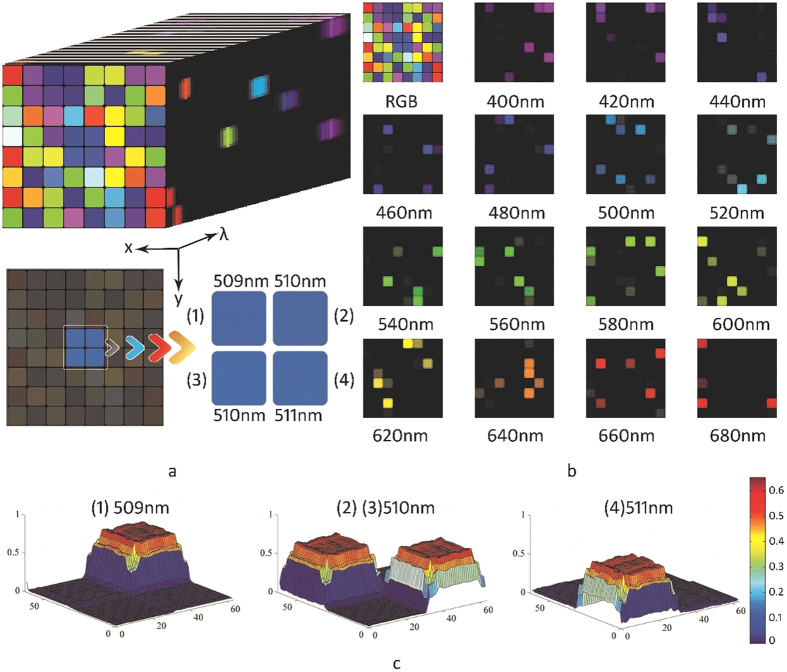
Reconstructed hyperspectral images of simulated data cube. (**a**) The simulated data cube consisting of 64 blocks, each has its own characteristic wavelength corresponding to the RGB colours. The wavelengths of the central four blocks are set at 509, 510, 510 and 511 nm, respectively. (**b**) The RGB image of the colour blocks and the pseudo-colour images reconstructed at different wavelengths from 400 to 680 nm at 20 nm intervals. (**c**) Demonstrates a 3-dimensional map of the reconstructed spectral images of the central four blocks at 509, 510 and 511 nm.

A novel mathematical model of HSI-SPFT was established; the logic of the specific simulation process is given by equations (1) to (9). To verify the feasibility of the mathematical model, a simulation was run via programming in MATLAB software; the results are shown in Fig. 1. First, a data cube (including 64 × 64 spatial pixels and 301 spectral channels) was constructed to simulate the measurable scene (see Fig. 1(a)). Both spatial information and spectral information were represented. Second, 2D measurement sequences were obtained via spatial projection and spectral modulation. Finally, the recovery data cube was acquired through the reconstruction algorithm. A total of 301 images were reconstructed, 15 of which are shown in Fig. 1(b). Because the spectral image of each channel was very sparse, the sampling rate was reduced to 6.25%. In other words, the image was accurately reconstructed by taking only 64 × 64 × 6.25% = 256 measurements (see the Methods section for the relevant procedures and principles). To illustrate the spectral resolution of the system, the four blocks at the centre have been reconstructed and analysed (see Fig. 1(c)). If the bandwidth is set to be narrow enough, then the centre wavelength of the adjacent blocks of 1 nm can be identified. The simulation results showed that the feasibility of the proposed approach, with the specific parameters required to be obtained experimentally.

### Experimental system setup and the results

**Figure 2:**
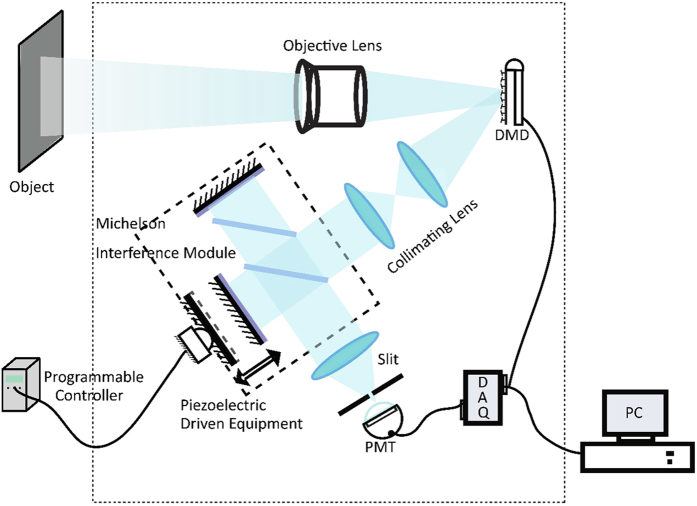
Schematic setup of the proposed Fourier transform hyperspectral imaging system based on single pixel technology. The object was imaged onto the DMD through an objective lens. After passing through the collimation lenses, the spatially modulated light was incident parallel to the rotating grating. Spectral information was acquired by the PMT and the data acquisition equipment.

The scheme of Fourier transform hyperspectral imaging based on the single pixel technology is shown in Fig. 2. In the experiment, the reflected light was incident on the DMD (V-7001VIS) through an imaging lens. The image covered as much of the coding region of the DMD as possible. The emergent light from the +12 degrees direction of the DMD was collimated into the Michelson interferometer, which is a core part of the Fourier transform spectral imaging system. The interferometer consists of a moving mirror, a fixed mirror and a beam splitter. To ensure high stability and precision, piezoelectric ceramic actuators are used to drive the moving mirror. As the voltage increases, the mirror moves at a nanometre scale, with movements guaranteed to be consistent. We set the step value of voltage at 0.001 V and the frequency at 1 kHz. The information of the interference pattern was then detected by the photoelectric conversion equipment (Photomultiplier Tube R5108, spectral response 400 to 1200 nm). The spectra were acquired by calculating the inverse Fourier transform of the collected data. The measurement values were assembled by selecting the corresponding values in each spectral curve, and then the spectral images were reconstructed accurately through equation (9).

**Figure 3:**
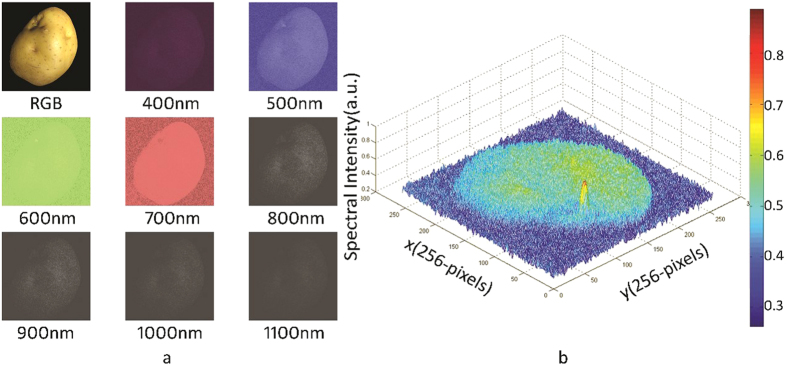
Reconstructed hyperspectral images of the potato at different wavelengths. (**a**) The RGB image captured by the standard camera and eight subfigures from the entire cube of 701 spectral channels at different wavelengths with pseudo-colour images and greyscale images, respectively. (**b**) The bud was distinguished from the normal part at the characteristic wavelength.

The reflection spectrum of an object has a “fingerprinting” effect^41^; thus, the reflection spectrum is typically used experimentally to verify the performance of the spectral imaging system. In the present study, the illumination source was a halogen lamp, and the light reflected from a potato was captured by the HSI-SPFT system. The images of the potato were reconstructed using an array of 256 × 256 pixels. At a sampling rate of 6.25%, 701 images were recovered from the hyper-spectral data cube at different wavelengths (Fig. 3). Figure 3(a) shows the image of the original target captured by a standard RGB camera and the reconstructed HSI-SPFT images from 400 to 1100 nm at 100-nm intervals.

Accurate spectral information was used to analyse the characteristics of the target. The shape and internal quality of the potatoes could be screened by analysing the hyper-spectral data cube captured by our system. Because of the different characteristics of the wavelengths, the normal part and the germination site of the potato exhibited different spectral intensities at different spectral bands. The position, shape, and size of the germinating part of the potato can thus be clearly observed. Therefore, potatoes can be screened non-destructively using HIS-SPFT (Fig. 3(b)).

## Discussion

We presented a hyperspectral imaging technique based on single-pixel technology and an interferometer. Experiments using reflection spectral imaging demonstrated the use of data compressibility in the system and its order of magnitude. Hyperspectral imaging systems have the potential for applications in fields that require a compact, accurate and fast imager with a high spectral resolution. The HSI-SPFT system could reduce the scanning time and produce higher quality images for the same scanning effort. However, our proposed technique has two limitations at present. First, the system must maintain a high level of stability to ensure the test signal is accurate. In the HSI-SPFT system, a piezoelectric transducer drives the moving mirror, providing a linear response with a jitter error of approximately 0.1%. We collected the interference fringes from a green LED with high symmetry, demonstrating that our HSI-SPFT system has good stability (see Supplementary material). Second, there is a trade-off between the integration time of the detector and the signal-to-noise ratio (SNR): a shorter integration time reduces the SNR.

In conclusion, a Fourier transform hyper-spectral imaging system based on single-pixel technology was presented in this paper. We applied our HSI-SPFT system to an experimental screening of potatoes using reflection spectral imaging. The system has a high resolution of 1 nm, as demonstrated by the calibration experiments, and a wide spectral range from 400 to 1100 nm for hyperspectral imaging. Although some problems still remain, we intend to improve the imaging performance of the HSI-SPFT system regarding image quality and thus its range of practical applications.

## Methods

### The compressive sensing model of the system

The basic principles of the HSI-SPFT approach are demonstrated through the following mathematical equations related to its spatial-spectral encoded sampling scheme. Our system is based on a Michelson interferometer and a single-pixel camera. As explained earlier, the FTHSI-SP technique provides a framework for the reconstruction of an n-dimensional vector, x, from m-dimensional linear measurements,


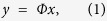


where *m* < *n* and *Φ* is the *m* × *n* sensing matrix. The underlying assumption is that x is sparse or has some sparse representation in some transformed domain. As the spatial modulator, the image was coded by the 0–1 state micro-mirror, which is controlled by the measurement matrix *Φ*. The measurement matrix is extracted from the transformation of the Hadamard matrix in our experiment. The 256 × 256-pixel image was reconstructed accurately at a 6.25% sampling rate, requiring m = 256^2^ × 6.25% = 65536 (n × 1) × 6.25% = 4096 measurements. The data (4096 frames) were randomly selected as the measurement matrix and were downloaded to the memory cell of the FTHSI-SP system to control the 0–1 state of the DMD. The signal, x, can be expressed in the spectral domain as:


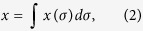


where *σ* is the wave number. Using equation (2), equation (1) can be expressed as:


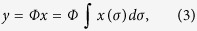


Because the matrix *Φ* is linear for the signal *x*, we have


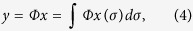


or


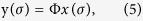


Thus, for the collimating modulated light incident to the Michelson interference system, each time the interferometry corresponds to a shift of DMD, it is equivalent to:


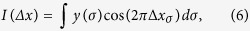


where *σ* is 1/λ, λ is the composite wavelength of the original image, and I(Δx) is the intensity of interference. This process modulates the spatial and spectral data. Therefore, we can acquire measurements as a function of the wavelength of the image after Fourier inverse transformation, *i.e*.,


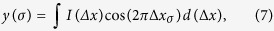


The reconstruction is performed using the TVAL3 algorithm^42^. TVAL3 is applied to this TV-based minimisation model in our framework as follows:


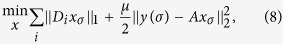


where *D*_*i*_*x*_*σ*_ is the discrete gradient vector of *x*_*σ*_ at position *i*, D is the gradient operator, and μ is a constant scalar that balances these two terms. According to equation (3), for three-dimensional reconstruction, the hyperspectral image is reconstructed according to


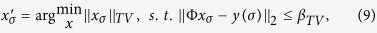


where *β*_*TV*_ is the regularisation parameter, and the *TV* norm of three-dimensional data is given in a supplementary file. Where *x*_*σ*_ is the spectral image for an original image at a wavelength of 1/*σ*.

### Simulation method and procedure

First, a three-dimensional data cube was constructed where the *x-y* dimension (64 × 64 pixels) represents the spatial information, and the third dimension (301 channels) represents the spectral information (Fig. 1). Figure 1a shows the cube consisting of 64 squares, each 8 × 8 pixels, with each block having its own characteristic wavelength. These characteristic wavelengths are set using Gaussian functions given by equation (1).


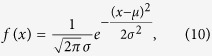


where *μ* is the centre wavelength, and 2*σ* is the full width at half maximun (FWHM). In addition to the four blocks at the centre position, the FWHM of the blocks are set to 10 nm, and their centre wavelength interval was set to 1 nm in the 400 to 700 nm spectral range. The centre wavelengths of the four blocks at the centre were set at 509, 510, 510 and 511 nm (Fig. 2b), and their FWHMs were all set at 1 nm. The blocks were then randomly arranged and coloured according to the projection of the centre wavelength of each block in RGB.

The data cube was then reconstructed as the original signal. Spectral images from each spectral channel were obtained by compression coding, spectral modulation and signal reconstruction. The measurement matrix was generated by the fast Walsh-Hadamard transform (FWHT). The inner product of the measurement matrix and signal matrix was used for spatial modulation. All these values were added to simulate the convergence of the lens to retain all the information. The Fourier transform of the measured value with spatial and spectral information was used to simulate the interference process. In the simulation sampling process, we have proposed two acquisition schemes. One scheme involves performing all the random measurements at each sampling point on the interference curve to obtain a data set of random values. The other scheme is based on sampling the complete interference patterns on each occasion of random measurement to obtain a data set of interference values. For the present study, we chose the second scheme. The power spectrum was then obtained using the inverse Fourier transform of each interference spectrum. The values of each power spectrum corresponding to the channel were then combined to provide the measured values. Finally, the spectral image of each channel was reduced to form a data cube by the reconstruction algorithm.

### Calibration

**Figure 4:**
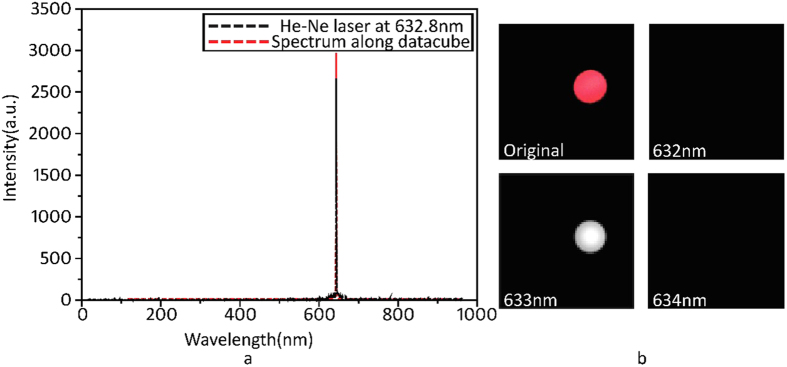
Reconstructed hyperspectral imaging of a laser spot. (**a**) The reconstructed spectrum from the data cube and the normalised spectrum measured by the spectrometer. (**b**) Shows the original image of the laser spot captured by a standard RGB colour camera and the reconstructed images at 632 nm, 633 nm and 634 nm using 12.5% of the measurements through spectral imaging.

To calibrate the spectral resolution of the proposed system, the spectra from a narrow spectral distribution light source (633 nm laser) were chosen for reconstruction. The 633-nm laser beam was expanded to a round spot then imaged onto the DMD. The normalised emission spectrum of the He-Ne laser red trace (Fig. 4) was measured directly by a spectrometer (Ocean QE65Pro) to a resolution of 1 nm. The spectrum (black line) was reconstructed using the data-cube in a spatial position where the laser spot was imaged (Fig. 4(a)). The reconstructed spectrum through the data-cube was found to perfectly match the measured spectrum. The cropped image of the laser’s spots was captured by a standard RGB colour camera (Fig. 4(b)). The other subfigures show the images reconstructed at 632, 633, and 634 nm. Compared to the left figure representing the reconstructed image of the laser spot, the right two figures show the absence of the relevant feature at that particular wavelength. Therefore, these images indicate the minimum spectral separation required for an image of the laser spot to be present or not present, demonstrating that the spectral resolution of the proposed system is 1 nm.

## Additional Information

**How to cite this article:** Jin, S. *et al*. Hyperspectral imaging using the single-pixel Fourier transform technique. *Sci. Rep.* **7**, 45209; doi: 10.1038/srep45209 (2017).

**Publisher's note:** Springer Nature remains neutral with regard to jurisdictional claims in published maps and institutional affiliations.

## Supplementary Material

Supplementary Materials
