# Supplementary material for: Hyperspectral imaging using the single-pixel Fourier transform technique
Source: Sci Rep. 2017 Mar 24;7:45209. doi: 10.1038/srep45209 (PMC5364546; doi:10.1038/srep45209)
Supplement: Supplementary Materials [file srep45209-s1.pdf]

Supplementary materials of
Hyperspectral imaging using the single-pixel Fourier transform technique

Senlin Jin, [1] Wangwei Hui, [1,*]Yunlong Wang, [1] Kaicheng Huang, [1] Qiushuai Shi, [1] Cuifeng Ying, [1] Dongqi Liu, [1] Qing Ye, [1] Wenyuan Zhou, [1] and Jianguo Tian, [1, 2]

[1]*The Key Laboratory of Weak-Light Nonlinear Photonics, Ministry of Education, Teda Applied Physics Institute and School of Physics, Nankai University, Tianjin 300071 , China*

[2]*jjtian@nankai.edu.cn*

[*]*hww@nankai.edu.cn*

1 Two schemes of data acquisition by HSI-SPFT

The following theoretical model of the HIS-SPFT has been proposed in the manuscript. As shown in Supplementary Fig. 1, the target is a scene with spatial(x-y) and spectral(λ) information. A data cube ($64 \times 64 \times 301$) was constructed first, which can be represented as,

$$\mathrm{A} = \int A(\sigma)d\sigma = \sum_{1}^{301} A_{\sigma}, \quad (1)$$

where σ is the wave number. The constructed data cube was coded by measurement matrix, the equation (1) can be expressed as,

$$\mathrm{y} = \Phi\mathrm{A} = \Phi \int A(\sigma)d\sigma, \quad (2)$$

where Φ is the measurement matrix. Then the modulated light is incident to the Michelson interference device in parallel, a set of the interference pattern corresponded to one state (0-1) of DMD, it is equivalent to,

$$\mathrm{I}(\Delta x) = \int y(\sigma)\cos(2\pi\sigma\Delta x)d\,\sigma, \quad (3)$$

where σ is $1/\lambda$, λ is the composite wavelength of the original image, and $\mathrm{I}(\Delta x)$ is the intensity of interference pattern. The whole process modulates the spatial and spectral information. In addition, two core components are time-varying in the system. One is the DMD, which shifts as time with a certain frequency. Another is the moving mirror, which moves as time at a certain speed. Therefore, we have two schemes to acquire the data. One is the DMD shifts once, the moving mirror moves a cycle. The frequency of DMD is 1 Hz, and the period of the moving mirror is one second. Another scheme is the moving mirror moves a set step, and DMD shifts a cycle. In the case, the step time of the moving mirror is 0.001s, and the frequency of the DMD is 1000Hz. Two schemes

have no difference through simulation, while the second scheme have a better result in the experiment. So the results were acquired by the second scheme in manuscript. And a function of the wavenumber can be acquired by Fourier inverse transformation.

$$y(\sigma) = \int I(\Delta x) \cos(2\pi\sigma\Delta x)\, d(\Delta x), \tag{4}$$

The reconstruction was carried out by the TVAL3 algorithm. The scheme of the data acquisition in HSI-SPFT is shown in Supplementary Fig .1.

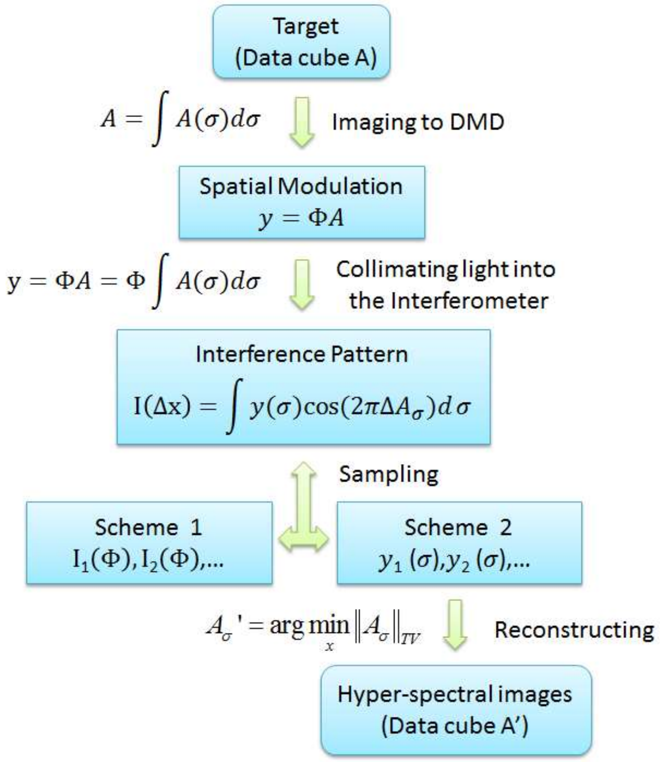

Supplementary Figure 1: **Two schemes of the simulation and experiment by HSI-SPFT.**

2 System specifications and performance analysis

2.1 System stability description and test

The performance is influenced by components in system, such as DMD (Texas Instruments 4100), interferometer, detector (PMT R5108) and ADC (NI USB 6211)[1,2]. The stability of the optics table was guaranteed first, and the optical path was also designed reasonably. The performance of the system was tested by a narrow band LED. The interference pattern was collected by the PMT as shown in Supplementary Figure 2(a). The performance of the system can be estimated by the periodicity and symmetry of the graph. The power spectrum of LED was obtained through the Fourier inverse transform. The SNR can further reveal the stability of the system. Supplementary Figure 2(b) shows the power spectrum of the LED, the SNR is 700:1, which shows the

high performance of the proposed system.

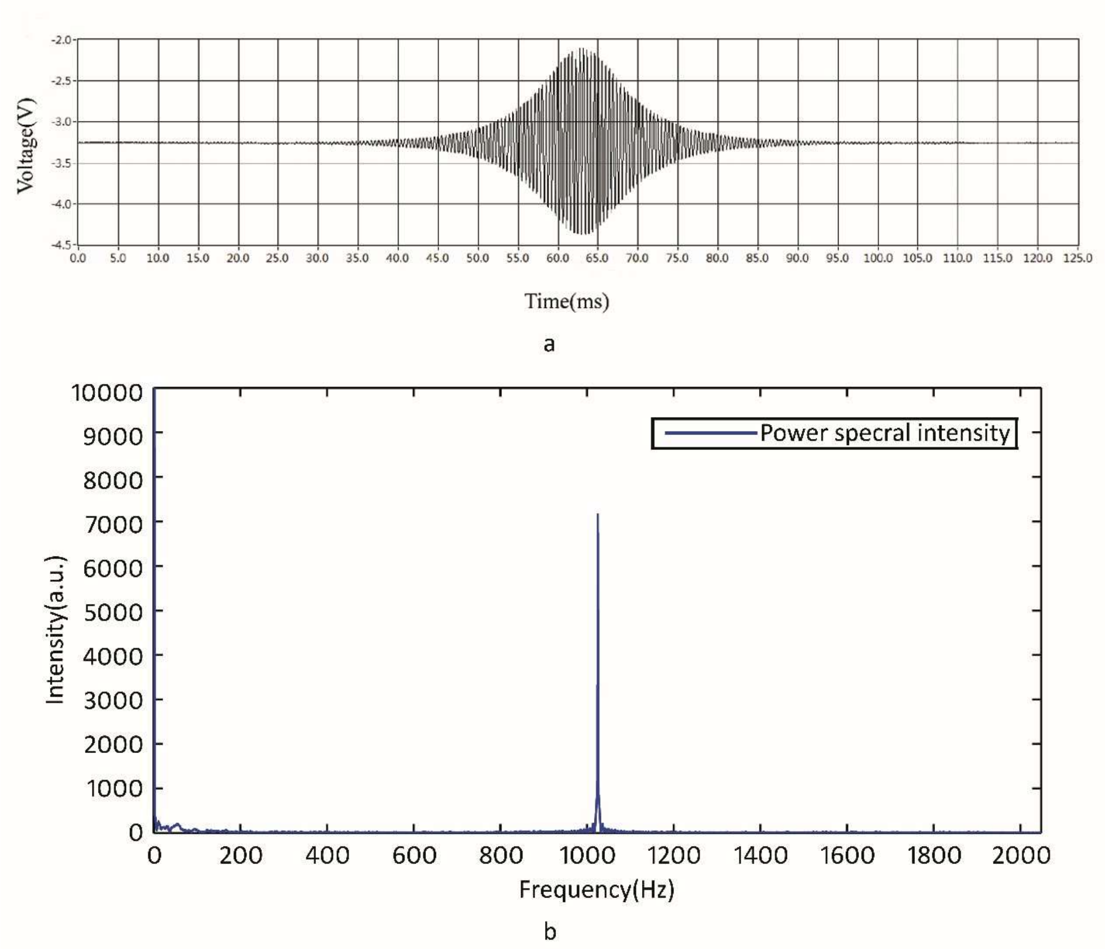

Supplementary Figure 2: **The interference pattern and the power spectral intensity of the narrow band LED at 480nm with 10nm FWHM.**

2.2 Multi-channel and High-throughput

Compared with the dispersive imaging spectrometer, Fourier transform imaging spectrometer has the advantage of multi-channel. If the spectral range from wavenumber ϑ_1 to ϑ_2 is measured with resolution $\delta\vartheta$ in time T, the number of spectral elements to be measured is,

$$N = {(\vartheta_2 - \vartheta_1)}/{\delta\vartheta}, \tag{5}$$

when measuring a target with a conventional dispersive spectrometer (typically with a slit), one exposure can measure only one spectral element. The total time of measurement is T, then the time corresponding to each spectral element is $\Delta T = T/N$. However, when using Fourier transform spectrometer, because the energy of all the spectral elements are simultaneously through the instrument and received the same time to form an interference pattern, so the measurement time for each element is $\Delta T' = T$,

which is the traditional dispersion N times. Since the noise in the detector output signal is random and independent of the signal level, the SNR of the receiver system is proportional to the square root of the measured time of each spectral element. Therefore, in a dispersion spectrometer, the SNR of the system is proportional to $\sqrt{T/N}$. In the Fourier transform spectrometer, the SNR is proportional to $\sqrt{T}$. Therefore, when the other conditions are the same, the same target is measured in the same time T, and the SNR of the Fourier transform spectrometer is $\sqrt{N}$ times higher than that of the dispersion spectrometer. Therefore, the advantages of multi-channel is also known as the advantages of high SNR.

Furthermore, if the spectral resolution is same, the Fourier transform spectrometer offers the advantage of high throughput compared to a dispersive imaging spectrometer. For the measured object, there is no slit limitation, the Fourier transform spectrometer can increase the incident light aperture, so its luminous flux is much larger than that of dispersion instrument.

2.3 Spectral resolution and Spectral range

In addition to multi-channel and high-throughput advantages, Fourier transform imaging spectrometers can accommodate a wide spectral range (limited by the spectral response of the detector and the nature of the optical material), while the spectral range of the grating imaging spectrometer is limited by its own principles. At present, the Fourier spectral transform technique do the best trade-off in spectral range and spectral resolution.

The spectral resolution and range of the HSI-SPFT is determined by the moving distance of the mirror and the number of sampling points. When the number of interference fringes is N, the distance of moving mirror is d, that is,

$$d = {}^{N\lambda}/_{2}, \quad (6)$$

where λ is the corresponding to the wavelength. Since the wave number (ϑ) is the reciprocal of the wavelength (λ), that is,

$$\vartheta = {}^{1}/_{\lambda}, \quad (7)$$

after differential,

$$\Delta\vartheta = {}^{|\Delta\lambda|}/_{\lambda^2}, \quad (8)$$

where $\Delta\vartheta$ is the resolution of wavenumber. For example, when λ is 400nm, the spectral resolution is 1nm， and the wave number resolution is 62.5 cm^{-1}. Or λ is 1100nm, the spectral resolution is 1nm， and wave number resolution is 8.26 cm^{-1}. And the wave number is defined as,

$$\Delta\vartheta = R = {}^{1}/_{(2L)}, \tag{9}$$

where the maximum optical path difference is L, wavenumber resolution is R According to the equation(8), (9)， when the spectral resolution is 1nm at 400nm， the minimum optical path difference is 80μm. When the spectral resolution is 1nm at 1100nm， the minimum optical path difference is 600μm. To avoid the loss of spectral information, reduce the spectral distortion and improve the computational efficiency, the sampling interval (Δx) is very important. The wave number range is set $\vartheta_{max} - \vartheta_{min}$, and the moving mirror uniform speed rate is V, the time is t, according to the equation (9), the motion time modulation frequency is,

$$f = 2V(\vartheta_{max} - \vartheta_{min}), \tag{10}$$

Based on Nyquist sampling theorem, the sampling frequency is

$$f_s = 2f, \tag{11}$$

According to equation (11), the minimum number of sampling points need to meet equation (12)

$$N_s = f_s t = 4V \times \vartheta_{max} t = 4L \times \vartheta_{max} = {}^{2\vartheta_{max}}/_{R}, \tag{12}$$

when the spectral resolution is 1nm at 400nm， the resolution of wave number is 62.5-cm^{-1}， the minimum sampling point N_s is 800.When the spectral resolution is 1nm at 1100nm， the resolution of wave number is 8.26 cm^{-1}， the minimum sampling point N_s is 6050.

3 Calibration

3.1 Translation stage accuracy test

In order to satisfy the above parameters, we chose the translation stage controlled by piezoelectric actuator (PI, P-62X) with feedback mechanism[3]. The positioning accuracy can meet the design requirements. The measurement results are shown in Supplementary Figure 3. Its close loop travel and resolution can reach the 800 μ m and 0.1nm, respectively. And the position accuracy is 0. 1%.

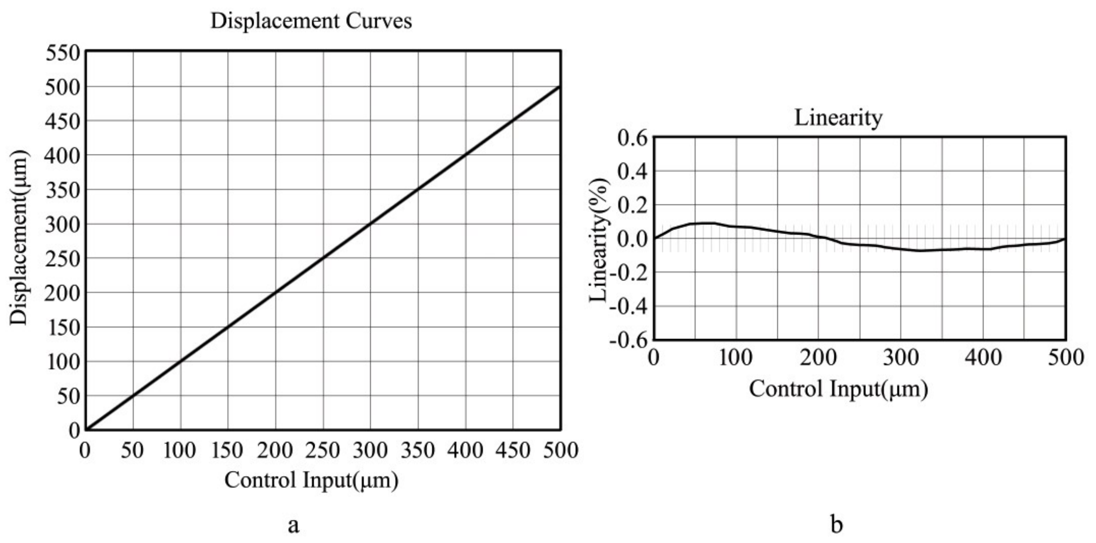

Supplementary Figure 3: **The interference pattern of the single wavelength LED.**

3.2 Spectral calibration

In the manuscript, the comparison of the result of the spectrometer and the result along the data cube. The ground truth was obtained by the spectrometer (Ocean optics QE65 Pro), whose spectral resolution is 1nm and spectral range is from 200nm to 1100nm. The specific index please refer to the reference (1). Supplementary Figure 4 shows the ground truth of the spectrum and the reconstructed bands of three LEDs at 418nm, 520nm, 622nm are obtained by the proposed system, respectively.

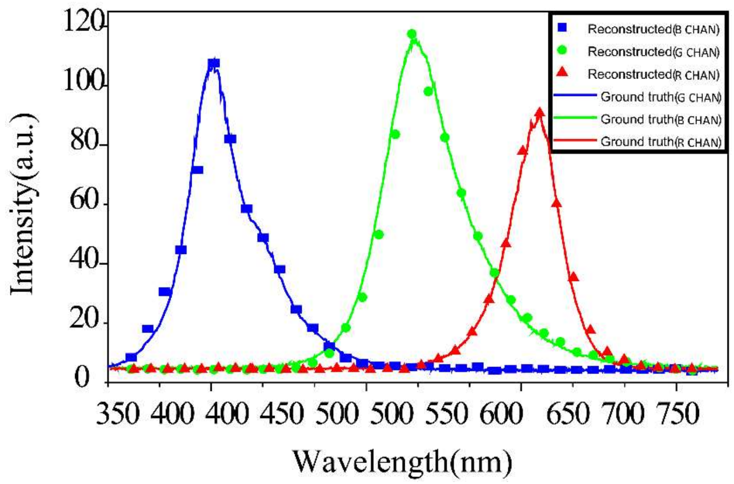

Supplementary Figure 4: **Spectral response ground truth of three LEDs compared with our method's reconstructed results.**

References

1 Ocean optics, spectrometer.

http://oceanoptics.com/support/technical-documents/qe65pro/
2 Hamamatsu, Photoelectric multiplier tube.
http://www.hamamatsu.com.cn/product/category/10079/0/10183/index.html
3 PI, PIHera Piezo Linear Stage
https://www.physikinstrumente.com/en/products/linear-stages-and-actuators/piezo-stages/p-6201-p-6291-pihera-piezo-linear-stage-202300/
